# Flood‐driven survival and growth of dominant C_4_ grasses helps set their distributions along tallgrass prairie moisture gradients

**DOI:** 10.1002/ajb2.16457

**Published:** 2025-01-08

**Authors:** Robert W. Wernerehl, Thomas J. Givnish

**Affiliations:** ^1^ State Botanist, Massachusetts Natural Heritage and Endangered Species Program Westborough 01581 MA USA.; ^2^ Department of Botany University of Wisconsin‐Madison Madison 53706 WI USA.

**Keywords:** ecological distributions, flooding tolerance, LD_50_, logit analysis, species sorting, water table depth

## Abstract

**Premise:**

Five C_4_ grasses (*Bouteloua curtipendula*, *Schizachyrium scoparium*, *Andropogon gerardii*, *Sorghastrum nutans*, *Spartina pectinata*) dominate different portions of a moisture gradient from dry to wet tallgrass prairies in the Upper Midwest of the United States. We hypothesized that their distributions may partly reflect differences in flooding tolerance and context‐specific growth relative to each other.

**Methods:**

We tested these ideas with greenhouse flooding and drought experiments, outdoor mesocosm experiments, and a natural experiment involving a month‐long flood in two wet‐mesic prairies.

**Results:**

*Bouteloua* promptly succumbed to inundation, so flooding intolerance likely excludes it from wet and wet‐mesic prairies. Competition is likely to exclude short‐statured *Bouteloua* from productive mesic sites. *Schizachyrium* is excluded from wet prairies by low flooding tolerance, demonstrated by all experiments. *Sorghastrum* had low flooding tolerance in both greenhouse and natural experiments, suggesting that physiological intolerance excludes it from wet prairies. *Spartina* had by far the greatest growth under the wettest mesocosm conditions; this and comparisons of species growth in monocultures vs. mixtures suggests that competition helps it dominate wet prairies. Indeed, quadrat presence of *Spartina* increased by 57% two years after flooding of two prairies, while that of upland grasses declined by 44%. The high flooding tolerance, lack of significant differences from other species in drought tolerance, and tall stature of *Andropogon* suggest that broad physiological tolerance combined with competitive ability allows it to thrive across the prairie moisture gradient.

**Conclusions:**

Flooding helps shape the distributions of dominant prairie grasses, and its effects may become more important as extreme rain events continue to increase.

Curtis (1959) classified tallgrass prairies of the Midwestern United States into five categories—dry, dry‐mesic, mesic, wet‐mesic, and wet—based on a compositional index (CI) based on five sets of indicator species, with the CI varying from 500 to 100 moving from dry to wet prairies. Curtis argued that compositional variation reflected gradations among sites in moisture availability, as affected primarily by soil texture, thickness, and depth to water table. Umbanhowar (1992) ordinated the data of Curtis and censuses from additional sites to conclude that Wisconsin prairies form a two‐dimensional continuum, with a first axis nearly identical to Curtis’ one‐dimensional gradient, and a second axis (explaining one third as much compositional variation as the first) tied to other edaphic differences, mainly among dry prairies on sand vs. shallow limestone vs. clay. Based on data for 126 prairies, the first axis was strongly correlated with soil water‐holding capacity (*r* = 0.80). Other studies throughout much of the tallgrass prairie biome in the United States—in Wisconsin (Partch, 1949, 1962), Illinois (Nelson and Anderson, 1983; Corbett and Anderson, 2006), Iowa and eastern Nebraska (White and Glenn‐Lewin, 1984), and Kansas (Heckathorn and DeLucia, 1994)—have documented a strong apparent effect on prairie composition by moisture supply or its likely correlates, based on variation in differences in overall topography, microtopography, depth to water table, soil texture, and subsoil permeability.

Wernerehl and Givnish (2015) demonstrated that Curtis’ prairie continuum does indeed parallel a gradient in soil moisture availability, based on variation across 17 prairie remnants in mean δ^13^C of leaf tissue for C_3_ plants, with CI being strongly correlated to δ^13^C (*r* = 0.59) and to scores on axis 1 of a Bray‐Curtis ordination of those sites (*r* = 0.89). Wernerehl and Givnish also found that soil fertility (as quantified by depth × cation exchange capacity) and soil mechanical impedance are even more strongly related to prairie composition than moisture availability.

Grasses with the C_4_ photosynthetic pathway dominate tallgrass prairies, account for most of their above‐ and belowground biomass, and help shape the conditions faced by the C_3_ grasses and forbs that compose the rest of such communities (Curtis, 1959; Tilman, 1989; Collins et al., 1998; Nippert and Knapp, 2007; McCain et al., 2011; Collins and Calabrese, 2012; Forrestel et al., 2015; Connell et al., 2019; O'Keefe et al., 2020). C_4_ grasses also differ from C_3_ grasses and forbs in their seasonality of photosynthesis and water use (Nippert and Knapp, 2007; Wang et al., 2013; Knapp et al., 2020) and so may be more likely to compete with other C_4_ grasses. In the Upper Midwest of the United States, the dominant grasses (Poaceae) in tallgrass prairies shift dramatically with position along Curtis’ moisture gradient, with short‐statured species like *Bouteloua curtipendula* (Michx.) Torr. and *Schizachyrium scoparium* (Michx.) Nash dominating thin, sandy, or otherwise dry soils and taller species like *Andropogon gerardi* Vitman, *Sorghastrum nutans* (L.) Nash, and *Spartina pectinata* Bosc ex Link dominating on progressively deeper, loamier, and wetter soils (Curtis, 1959; Henderson, 1998) (Table 1). (Nomenclature follows the USDA Plants Database [NRCS, 2024]). Calculations based on species frequencies in different kinds of prairies and assigning ecological scores of 1 to 5 to dry through wet prairies, respectively, yield average positions on the moisture gradient of 1.52 for *Bouteloua*, 2.07 for *Schizachyrium*, 3.14 for *Andropogon*, 3.62 for *Sorghastrum*, and 4.67 for *Spartina* (Table 1). The same distributions of these species along a topographic moisture gradient can be seen at the Konza Prairie Biological Station (KPBS) in eastern Kansas, but with *Bouteloua gracilis* substituting for *B. curtipendula* on the driest sites (Heckathorn and DeLucia, 1994).

**Table 1:** Percentage of 1‐m^2^ quadrats (or sites*) in which the five dominant C_4_ grasses occur in Wisconsin tallgrass prairies as a function of position along Curtis’ dry‐wet prairie continuum (Henderson, 1998). Data are from 247 stands studied from 1940 to 1958. Average positions are calculated here, based on fractions of occurrences in each kind of community and community position, scored from 1 to 5 for dry to wet prairies.

Species	Dry (1)	Dry‐mesic (2)	Mesic (3)	Wet‐mesic (4)	Wet* (5)	Average position
*Bouteloua curtipendula*	60	64				1.52
*Schizachyrium scoparium*	75	66	21	34		2.07
*Andropogon gerardi*	46	52	35	32	73	3.14
*Sorghastrum nutans*	2	10	18	21	18	3.62
*Spartina pectinata*			7	12	96	4.77

The causes of the different distributions of dominant C_4_ grasses along the tallgrass prairie moisture gradient are unknown. It is remarkable that, more than 50 years after being documented by Curtis (1959), the primary axis of local variation in tallgrass prairie composition in the Upper Midwest and specifically the distribution of its most dominant species remain unexplained (Wernerehl and Givnish, 2015). Although ecological and physiological studies have been conducted on some of these species, most have focused on a single species (e.g., Giuliani et al., 2014; Bachle and Nippert, 2022), just two or three species (e.g., Lemoine et al., 2017; Lemoine and Budny, 2022; Dekirmenjian et al., 2024), or have not connected physiological differences to differences in growth, survival, or ecological performance (e.g., Hoffman et al., 2018), and so do not answer the critical question of how the relative survival and competitive ability of all five dominant species shift along environmental gradients. The closest approach to such illuminating results to date might be that of Heckathorn and DeLucia (1994), who showed that the rank order of *Spartina*, *Sorghastrum*, *Andropogon*, *Schizachyrium*, and *Bouteloua gracilis* along the topographic moisture gradient at KPBS precisely matches their rank order for the leaf water potential at which their stomata close completely and net photosynthesis ceases, at –3.5, –5.5, –6.5, –7.5, and –8.0 MPa, respectively. These results point to species that dominate drier prairies being more drought tolerant. Such differences must ultimately, however, be tied to differences in survival and competitive ability at different points along an environmental gradient.

Potential causes of the differential distribution of dominant C_4_ grasses along local gradients of moisture availability in prairies are numerous and could include variation among species in physiological tolerance to different conditions or in context‐specific competitive ability (Ellenberg, 1953; Tilman, 1988; Smith et al., 2023), susceptibility to grazers (Weatherford and Myster, 2011; Collins and Calabrese, 2012), attack by pathogens (Mitchell et al., 2002; Cox et al., 2013; Dendy et al., 2017) or association with mutualists (Hartnett and Wilson, 1999; Weremijewicz et al., 2016; Wang et al., 2019). Here we identify three general hypotheses that might account for the differential distribution of dominant prairie grasses based on their physiological tolerance and context‐specific competitive ability:

(1) **Species dominate portions of gradients where they have an advantage in whole‐plant growth measured in monoculture.** This theory traces back at least to Ellenberg (1953), underlies the optimality modeling approach used by many ecologists, and has proven highly informative for traits affecting light capture and carbon gain (e.g., Givnish, 1979, 1986, 2002; Schwinning and Ehleringer, 2001; Mäkela et al., 2002; Givnish and Montgomery, 2014; Smith et al., 2023). In accord with the Ellenberg hypothesis, McAllister et al. (1998) showed that plant cover for 16 core species at KPBS increased significantly with average photosynthetic rate. That study, however, did not explain why different species shift in abundance along topographic gradients. Growth timing can be as important as growth rate. In a California salt marsh, Bonin and Zedler (2008) showed that, among three species showing equal productivity in field monocultures, the locally dominant species was the one that grew tallest first and intercepted the most light.
(2) **Species dominate portions of gradients where they can deplete limiting nutrients more than other species and still survive, as measured in monoculture.** This formulation is due to Tilman (1980, 1982) and traces to a model by Rosenzweig and MacArthur (1963) for competition between predators that share a prey species. Species that require lower levels of a given limiting resource to sustain themselves should ultimately displace other competitors. Known as R* theory, this approach has stimulated a great deal of research. Dybzinski and Tilman (2007) confirmed several predictions of this theory with an 11‐year experiment in which they grew six prairie species in monoculture and mixture and quantified their minimum requirements for nitrate (N*) and light at ground level (I*) as a function of total soil N. Surprisingly, there have not yet been any tests of R* theory using water as a limiting resource.
(3) **Species dominate portions of gradients where they can better survive flood or drought, as measured in monoculture.** Differences in physiological tolerance might exclude species from different portions of the dry–wet prairie continuum. If flood or drought tolerance is inversely related to competitive ability under more mesic conditions, this hypothesis suggests that species should replace each other along a gradient of decreasing moisture availability in order of flood tolerance (or reverse order of drought tolerance).

These hypotheses span the range of simple mechanisms by which differences among dominant species in physiological tolerance and competitive ability are likely to shape each other's distributions along gradients. Tests of the third hypothesis—involving the role of physiological tolerance in limiting species distributions—are straightforward and a necessary first step toward testing the first two hypotheses, which are based on different mechanisms of competition. Here we conducted a series of flooding studies on the C_4_ grasses that dominate different portions of the wet–dry tallgrass prairie continuum, including a greenhouse experiment to test hypothesis 3, an outdoor mesocosm experiment initially designed to test hypotheses 1 and 3 (involving physiological tolerance and competitive ability in different contexts), and a natural experiment created by a month‐long flood in two wet‐mesic prairies to test hypothesis 3. We tested the idea that species dominance at different points on the prairie continuum is determined, at least in part, by their ability to survive flooding. A limited test of hypothesis 1—that relative growth rates, as measured in monocultures, help determine species distributions—was also afforded by the mesocosm study.

## MATERIALS AND METHODS

Our greenhouse, mesocosm, and field studies focused on the C_4_ grasses *Bouteloua curtipendula*, *Schizachyrium scoparium*, *Andropogon gerardii*, *Sorghastrum nutans*, and *Spartina pectinata* (Poaceae), which dominate dry, dry‐mesic, mesic, wet‐mesic, and wet prairies, respectively, in many parts of the tallgrass prairie biome. Phylogenetically unstructured statistical analyses of survival and growth are used throughout this paper. We hypothesized that competitive success should depend on whether a species has significantly higher rates of survival or growth, not on how closely related they are; unstructured tests are justified for evaluating such hypotheses.

1. **Flood‐to‐death greenhouse experiment.** Seedlings of four native prairie grasses (*Bouteloua curtipendula*, *Schizachyrium scoparium*, *Andropogon gerardii*, *Sorghastrum nutans*) were grown in two well‐ventilated greenhouses. Attempts to germinate seeds of *Spartina pectinata* from several sources failed. *Bouteloua* and *Schizachyrium* are dominants in dry and dry‐mesic prairies (Table 1) and were considered indicators of dry prairies by Curtis (1959). *Andropogon* is abundant across the dry–wet continuum; *Sorghastrum* is especially common in the mesic to wet portion of that gradient; and *Spartina* is an indicator of wet prairies (Table 1; Curtis, 1959) Temperatures were held at 25.5°C for the 13‐h photoperiod when supplemental light was supplied, then dropped to 17.7°C at night. Hourly measurements of air temperature were taken in both greenhouses (8092 measurements during the experiment in both houses). Greenhouse 1 was slightly but significantly cooler than greenhouse 2 (21.5 ± 3.3°C vs. 21.7 ± 3.7°C). All species were grown in flats from seeds of local genotypes from Ion Exchange Nursery (Harpers Ferry, IA, USA), sourced from different prairies averaging about 100 miles from Madison, Wisconsin. Seedlings were transplanted after 3 weeks to deep pots (20 cm tall × 16.5 cm diameter) to permit extensive root growth. For each species, two individuals were planted per pot, each 4 cm from the pot edge and 8.5 cm from each other. The potting mix consisted of a 3:1 mix of sand and silt loam, composited from the same bulk supply. A total of 200 pots were prepared per species, with half randomly assigned to each greenhouse. Pots were well‐watered for 3 months before entering treatment, with 70 pots per 122 cm × 183 cm wire‐mesh bench.

Each bench was rotated every few days counterclockwise, both on its own axis and around the greenhouse, so that plants were exposed to all conditions within the greenhouse as they developed. In addition, plants were rotated within each bench, with all edge plants swapped to the middle and vice versa. Plants were placed in the flooding treatment when about to flower.

### Flooding treatment

Eight boxes were placed on top of the greenhouse benches, double‐lined with plastic, and filled with water. For each species, 55 pots per box were then placed so that the water level was 5 cm above the soil. A total of 90 pots from each species were placed in the flood treatment for up to 33 days. Beginning on the fifth day, and thereafter every fourth day, 10 pots of each species were removed from the flood treatment, tagged, and then watered daily with free drainage until 10 days beyond the end of the flooding experiment. The flooding treatments thus lasted 5, 9, 13, 17, 21, 25, 29, or 33 days.

### Visual scoring assessment

At 6 days after the last pots were removed from treatment, the area of functional leaf tissue was visually estimated for all treatment and control pots. Plants were scored from 1 (no functional leaf tissue) to 10 (all functional leaf tissue). To be considered functional, a leaf had to be medium to dark green and flexible, not brittle. Scores associated with particular times in treatment therefore reflect the amount of time plants were held in treatment, as well as the complementary period in which they were held under less stressful conditions. Tissue score data are provided in Dryad file Fld2.csv (https://doi.org/10.5061/dryad.m37pvmd8t).

### Data analyses

Tissue survivorship was related to time in treatment for both experiments using logit analysis. The score *S*_*ijk*_ for all individuals (*k* = 1 to *N*) of species *i* on the *j*th date for a given experiment was first transformed into an estimate of proportion green tissue remaining, *P*_*ijk*_ = (*S*_*ijk*_ – 0.5)/10, mapping the (0, 1) interval onto (0.05, 0.95). Then we calculated the logit‐transformed rating, *Y*_*ijk*_ = ln (*P*_*ijk*_/(1 – *P*_*ijk*_), which maps the (0, 1) interval onto (–∞, +∞). If *D*_*j*_ is the number of days in treatment for the *j*th date, and *m* = 1 or 2 indicates the greenhouse used, then the model for logit analysis is

(1) $$Y_{ij}=(\alpha_{i}+a_{im})+(\beta_{i}+b_{im})D_{j}+\varepsilon_{j},$$

where *α*_*i*_ and *β*_*i*_ are the intercept and slope for species i, and *a*_*im*_ and *b*_*im*_ are the random perturbations to the intercept and slope for each species j and greenhouse m. We assume that (*a*_*im*_, *b*_*im*_) ≈ iid N ((0, 0), ∑), meaning that each of the eight pairs are independent draws from a joint bivariate normal distribution with mean 0 for each parameter and a variance–covariance matrix ∑. We also assume that *ε*_*j*_ ≈ iid N (0, σ^2^). Note that, while the *Y*_*ijk*_ ratings are, in principle, drawn from a continuous distribution on (–∞, +∞), in practice they could only assume 10 discrete values.

After obtaining the best model for each species and treatment using Eq. 1 (R code kindly provided by Bret Larget and Jason Carmignani and run in RStudio version 2023.03.1 + 446 [RStudio Team, 2019]; see doi:10.5061/dryad.m37pvmd8t), we then solved for the number of days D at which the curve fell below zero on the transformed data, corresponding to an original score of 5.5, the midpoint of the interval (1, 10). In essence, D corresponds to the LD_50_ for flooding—that is, the “lethal dose” (days of flooding) required to kill 50% of live tissue. Given that all plants were in good condition at the outset of the flooding treatment and expected to be in poor condition at the end of that treatment, the time required to inflict apparent loss of function on half the leaf surface—LD_50_—is a useful index for comparing species. Estimates of the mean, standard error, and 95% confidence intervals of LD_50_ for each species and experiment were calculated in R (R Core Team, 2013). Tukey's honestly significant difference (HSD) test, implemented in JMP 10 (2012; SAS Institute, Cary, NC, USA), was used to determine which species differed significantly in LD_50_.

2. **Mesocosm study of growth vs. depth to water table**. We measured aboveground growth of the five dominant C_4_ grass species planted in monocultures and a five‐way mixture at five different water‐table depths in metal drums. Our initial plan was to measure the relative growth rate and survival of each species as a function of water‐table depth. Seeds of each species were obtained from Prairie Nursery (Westfield, WI, USA), germinated in a greenhouse, and transplanted after 20 d into flats with cells 8 × 8 × 7.5 cm deep. After 2 months, the resulting plugs were transplanted outdoors in mid‐June 2007 to food‐grade, enamel‐lined, 220‐L drums (82.5 cm tall × 57 cm diameter; Pure Sweet Honey, Verona, WI, USA), in an open field at the West Madison Agricultural Station (Verona, WI). Planting substrate was a 3:1 mixture of torpedo sand and local silt loam. This substrate was thoroughly homogenized with a portable hammermill composting mixer and then dumped into the drums using a front‐end loader and filled to within 2.5 cm of the upper lip.

A total of 150 drums were placed in a grid of 12 rows × 12 columns, with an additional row of 6 drums; drum edges were set 1 m apart. Five water‐table treatments were assigned to drums by random‐number table. Treatments were enforced by punching rows of eight drainage holes (1.5 cm wide) evenly spaced at 0, 20, 40, or 60 cm from the bottom of drums, thus creating the five different depths to the water table of 0, 20, 40, 60, and 80 cm below the surface. These depths correspond to treatments 1 through 5. Test wells of ¾‐inch PVC pipe were driven into the middle of each drum. Holes were drilled in the bottom of the PVC pipe, a cotton sock placed over them, and 20‐cm long, 1¼‐inch PVC pipe with header and numerous holes placed over that assembly. Wells were capped loosely to prevent evaporation. Water depth was measured at intervals using a dipstick, with depth adjusted to compensate for dipstick displacement.

Five plugs were planted in monocultures for each species for five water‐level treatments with five replicates per treatment, for a total of 25 drums per species, 125 barrels of monocultures overall, and 125 plugs of each species grown in monoculture. Drums with mixtures had one transplant of each species, planted in random circular order, for the same number of treatments and replicates, resulting in 25 drums with mixtures and 25 transplants of each species in mixtures. All drums were planted in a circular pattern, with transplants placed with an equal distance between each neighbor and the mesocosms edge; a form was used to ensure duplication of placement across mesocosms. Mesocosms were well‐watered for 1 month to permit full establishment of the transplant, with water‐level treatments beginning in mid‐July.

Torrential rains throughout late summer 2007 resulted in the experimental substrates being saturated or even inundated, converting this experiment into an unplanned study of growth and survival under damp to flooded conditions. During August 2007 alone, Madison officially recorded 38.6 cm of rainfall, which was 27.6 cm above the long‐term average. It set all‐time records for the wettest August and the wettest month overall at Madison (NOAA/National Weather Service, Milwaukee/Sullivan, WI, USA). In addition, June 2008 was the wettest June on record.

Total aboveground tissue for each plug was harvested after cessation of growth (or death) in early November 2007 and dried at 50°C for 4 days before weighing. Mean ± SD aboveground biomass was calculated across individuals for each species for each nominal water‐table depth for the monoculture treatment and separately for the mixtures. Means and standard deviations were also calculated for individuals pooled across water‐table depths for each species. We used Tukey's HSD test to determine which differences in biomass among species were significant, with monocultures and mixtures considered separately. Significance of differences between biomass in monocultures vs. mixtures was assessed for each species using a two‐tailed *t*‐test. Raw data are provided in Supplemental Table S1 (https://doi.org/10.5061/dryad.m37pvmd8t). An ANOVA of biomass as a function of species and nominal water‐table depth (treatments 1–5, see above) was conducted after the data were square‐root transformed to ensure normality. Regressions of aboveground biomass against nominal water‐table depth were calculated for each species in monoculture, after transformation of variables if needed to ensure linearity; we conducted an analysis of covariance (ANCOVA) to determine whether any of the lines differed significantly in slope.

**(3) Comparative data: resurvey for flood effects**. In 2007, two wet‐mesic prairies (Faville and Snapper) in the floodplain of the Crawfish River in Jefferson County, Wisconsin, were chosen for study. Both were dominated by native prairie plant species with little invasion by exotics. These sites were censused along transect lines in June 2007. Heavy rains (142 mm) on 25 August 2007, resulted in a 5‐year flood (peak flow ca. 90.5 m^3^ s^–1^) on the Crawfish River at Milford (data downloaded from http://usclimatedata.com and https://nwis.waterdata.usgs.gov). Even heavier rains (265 mm) between 5 and 13 June 2008 resulted in an even greater flood (ca. 204 m^3^ s^–1^)—the largest during the 33 years between 1990‐2023—and covered both prairies with floodwaters for ca. 28 days in June and early July 2008. These sites were recensused along the same transect lines in June 2009 to evaluate the apparent impacts of prolonged flooding.

At each site, five parallel 50‐m transects were established at intervals of 5 to 10 m; transects were located to minimize any apparent variation in slope, slope aspect, soil texture, soil depth, and depth to water table. The presence/absence of all graminoid species was noted in 0.25 × 0.25 m quadrats placed randomly within each of ten 5‐m segments of each transect, providing a stratified random sample. The small size of the quadrats permitted each to be inspected closely to identify all graminoid species, including those not in flower or fruit. All vascular plant species occurring within 10 m of each transect at a site were also tallied. We calculated increases and decreases between 2007 and 2009 in the number of quadrats occupied by individual graminoid species, testing each for significance using a *χ* ^2^ test. We also compared the change in frequency of *Spartina pectinata*—an indicator species for wet prairies—vs. the pooled change in all upland graminoid species (i.e., those most common in dry, dry‐mesic, or mesic prairies), and again tested for a significant difference using a *χ* ^2^ test. Finally, for each prairie, we calculated a measure of flooding growth for the four focal species based on the ratio of quadrats occupied in 2009 vs. 2007; we calculated the average of those scores across prairies and ranked the species based on those averages. *Bouteloua* was a priori considered to be the least tolerant of flooding based on its absence from both prairies on both dates.

**Figure 1:**
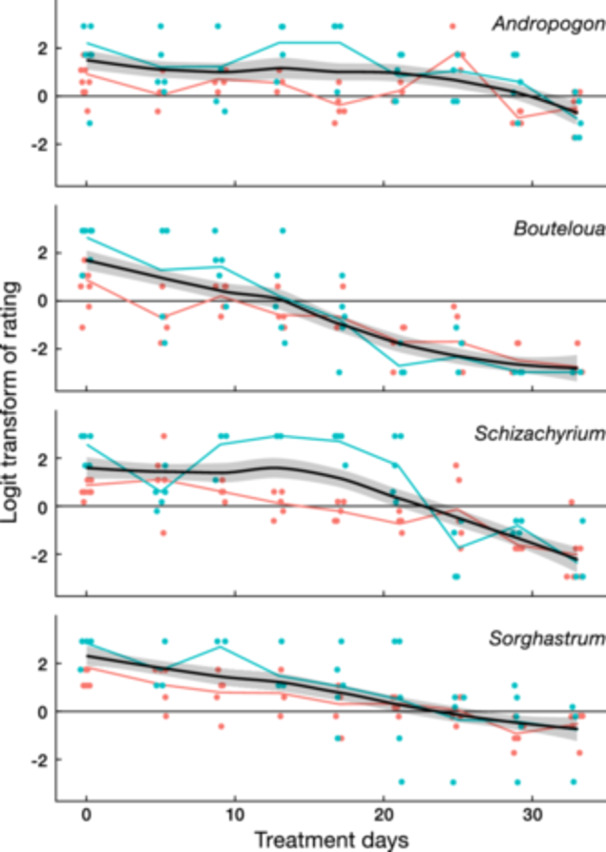
Logit analysis of average tissue survival after flooding for different durations. Horizontal lines are equivalent to a visual rating of 5.5. Thick curves are locally smooth regressions; the grey ribbon indicates the 95% CI. LD_50_ values correspond to where the regressions intersect the green lines and represent the number of days of flooding required, on average, for 50% of leaf tissue to die. Reddish‐orange dots and lines are pots from greenhouse 1; turquoise dots and lines are from greenhouse 2. Species are indicated along the right‐hand edge.

**Table 2:** LD_50_ values (mean ± 95% CI) based on logit analyses for tolerance of flooding (see Figure 1). Significant differences in LD_50_ are indicated by non‐overlapping superscripts.

Species	LD_50_
*Bouteloua*	11.2 ± 7.0^A^
*Schizachyrium*	19.5 ± 6.0^AB^
*Sorghastrum*	24.3 ± 5.4B^C^
*Andropogon*	31.4 ± 10.4**^C^**

## RESULTS

(1) **Flood‐to‐death greenhouse experiment.** Logit analysis of the flooding experiment analysis demonstrated that tissue survival of all four tested species declined through time, starting at a projected logit score of ca. 2.0 and ending at a projected score of –1.5 (Figure 1). *Bouteloua* was significantly more flooding‐sensitive (LD_50_ = 11.2 ± 7.0 days) than all other species, and *Andropogon* was significantly less sensitive (LD_50_ = 31.4 ± 10.4 days) than all other species except *Sorghastrum* (Table 2). *Schizachyrium* was significantly less flooding‐tolerant than *Andropogon* and significantly more flooding‐tolerant than *Bouteloua*. An ANCOVA showed a significant greenhouse effect (*P* < 0.0021), indicating that the greenhouses were not equivalent to each other in effects on the grass species. Visual inspection of the data indicated that *Andropogon* and *Schizachyrium* were the only species with a noticeable greenhouse effect; both remained in good condition slightly longer in the slightly cooler greenhouse (21.5° vs. 21.7°C), where adverse effects might be expected to slow due to lower metabolic rates. The same rank of flooding tolerance across species was maintained in both houses.
(2) **Mesocosm study of growth vs. depth to water table**. Final biomass differed significantly among species in monoculture, pooled across nominal water‐table depths, with interspecific differences accounting for 26% of the overall variance (*P* < 0.00001 for ANOVA; F ratio = 62.73 for 620, 4 df; Supplemental Table S2). Under the extremely wet conditions of late summer 2007, *Spartina* had the highest average biomass, significantly greater than that of *Andropogon*, which in turn had significantly greater biomass than *Bouteloua*; *Andropogon*, *Schizachyrium*, and *Sorghastrum* did not differ significantly in biomass (Table 3). That is, the nominal dominant of wet prairies performed best; the nominal dominant of dry prairies did worst; and the remaining species showed intermediate growth. The data from the plants growing in mixture mesocosms showed that *Spartina* had a significantly greater biomass than the other species and that the other species did not differ significantly from each other (Table 3). All species except *Spartina* had less biomass in the mixture than in the monoculture, indicating greater inter‐ vs. intraspecific competition; significance of the monoculture vs. mixture differences for those species ranged from 0.5% to 15.3%.

**Table 3:** Mean (± SE) of aboveground biomass (g) per individual for each species in the mesocosm monocultures (*N* = 125 per species) vs. mixtures (*N* = 25 per species), pooled across water‐table treatments. Note the identical rankings of species performance in monocultures and mixtures. Significant differences between species indicated by non‐overlapping superscripts; tests based on square‐root‐transformed data using Tukey's HSD. Significance of differences between biomass in monocultures vs. mixtures was assessed using a two‐tailed *t*‐test. Note that all species show less biomass in mixture vs. monoculture except *Spartina*, pointing to a greater impact of interspecific vs. intraspecific competition, with the former presumably reflecting interactions with growth‐dominant *Spartina*.

Species	Monoculture	Mixture	Significance (mono vs. mix)
*Spartina*	31.53 ± 1.41^A^	34.21 ± 4.42^A^	0.808
*Andropogon*	20.35 ± 0.81^B^	15.27 ± 1.37^B^	0.005
*Schizachyrium*	18.02 ± 0.68^BC^	15.05 ± 1.46^B^	0.068
*Sorghastrum*	16.30 ± 0.71^CD^	13.88 ± 1.61^B^	0.153
*Bouteloua*	14.20 ± 0.84^D^	11.40 ± 1.43^B^	0.099

**Figure 2:**
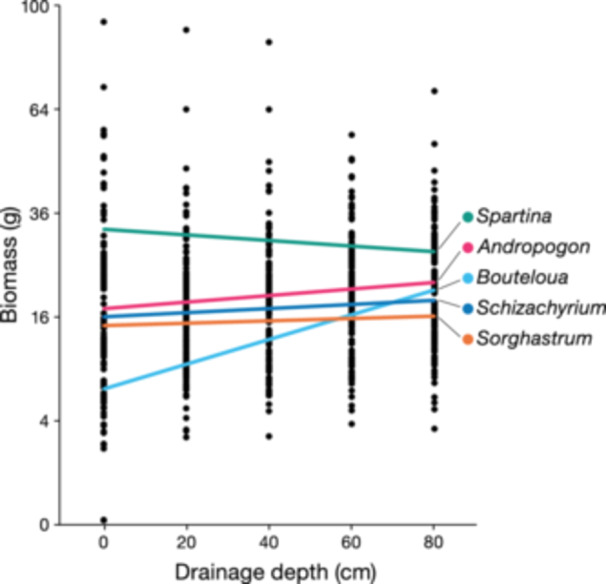
Linear regressions of square‐root‐transformed aboveground biomass as a function of water‐table depth for *Andropogon gerardii* (A), *Bouteloua curtipendula* (B), *Schizachyrium scoparium* (C), *Sorghastrum nutans* (O), and *Spartina pectinata* (P) grown in monoculture in mesocosms. Based on ANCOVA, regression slopes are significantly different for *Spartina* vs. *Bouteloua* (*P* < 0.0001).

As expected, the greater difference in biomass between the nominal dominants of wet and dry prairies was achieved in the wettest treatment (i.e., that with the shallowest drainage holes and shallowest nominal water‐table depth [0 cm]), where *Spartina* had significantly greater final biomass than *Bouteloua*, with both also differing significantly from the other three species (Figure 2, Table 4). Based on informal observations during the study, most of the *Bouteloua* plants appeared to have died during the severe August flooding. *Spartina* biomass decreased linearly with treatment level (1–5, corresponding to drainage hole depths and nominal water‐table depths of 0–80 cm):

(2) $$Spartina\;{\text{biomass}}^{0.5}=5.78\;-\;0.106\;\times \;\text{Treatment}\;\text{level}$$

(*r* ^2^ = 0.013, *P* = 0.055 for 123 df). A regression against ln Treatment level was significant (*P* < 0.03), but the variance explained was still quite small (*r* ^2^ = 0.03). By contrast, *Boutleloua* biomass increased significantly and substantially with treatment level:

(3) $$Bouteloua\;{\text{biomass}}^{0.5}=2.14+0.47\;\times \;\text{Treatment}\;\text{level}$$

(*r* ^2^ = 0.29, *P* < 0.0001 on 123 df; Figure 2). *Bouteloua* showed an even stronger, plateauing response to ln Treatment level (*r* ^2^ = 0.36). An analysis of covariance (ANCOVA) fitting both *Bouteloua* and *Spartina* linearly to treatment level found that the two regression slopes differed in highly significant fashion (*P* < 0.0001). *Andropogon* biomass also increased significantly toward the driest treatment (*P* < 0.04), with regressions for *Schizachyrium* and *Sorghastrum* closely paralleling *Andropogon* but not varying significantly with nominal water‐table depth (Figure 2). A two‐way ANOVA showed significant effects of species, water‐table depth, and an interaction between both, with the variance explained climbing to 38% (see Supplemental Table S2).

**3. Natural experiment: prairie resurveys for flooding effects.** Among our focal species, frequencies of *Schizachyrium* and *Sorghastrum* declined significantly with flooding at both Faville and Snapper Prairies, with *Schizachyrium* essentially disappearing, and *Sorghastrum* losing 74–100% of its original incidence (Table 5). *Andropogon* was unchanged at Snapper but increased from 24% to 58% cover at Faville. *Spartina* increased (albeit nonsignificantly) at both sites, though its 3‐fold proportional increase at Faville was greater than that of *Andropogon. Bouteloua* was absent from both prairies before and after the 2008 flooding event. Among other grasses present, *Sporobolus heterolepis* (A. Gray) A. Gray decreased sharply and significantly at both sites after flooding, as did *Agrostis gigantea* at Snapper (Table 5). Among sedges (Cyperaceae), unidentified species of *Carex*, *Eleocharis compressa* Sull., and *Calamogrostis canadensis* (Michx.) P. Beauv.—all characteristic of wet prairies or even wetter sedge meadows—and *Digitaria cognata* (Schult.) Pilg. from drier sites showed highly significant increases in coverage following flooding. There was a highly significant increase in the frequency of *Spartina* vs. all upland grasses after flooding, with *Spartina* increasing by 57%, while upland grasses decreased by 44% (*χ* ^2^ = 8.74, *P* < 0.0032 for 1 df), with the ratio of frequencies nearly tripling, from 6.5% to 18.2%. The contrast is more extreme if the weedy, short‐lived *Digitaria*—presumably invading bare microsites caused by flooding—is excluded. Under that assumption, upland grasses decreased by 77% (*χ* ^2^ = 31.9, *P* < 10^−6^) and the relative frequency of *Spartina* increased from 6.5% to 44.9%—roughly a 7‐fold increase.

**Table 4:** Mean aboveground biomass per individual for each species in the mesocosm monocultures (*N* = 25 per species) for the wettest treatment. Significant differences between species indicated by non‐overlapping superscripts corrected using Tukey's HSD.

Species	Mean biomass ± SE (g)
*Spartina*	39.91 ± 3.68^A^
*Sorghastrum*	17.36 ± 1.51^B^
*Schizachyrium*	17.29 ± 1.68^B^
*Andropogon*	17.02 ± 1.20^B^
*Bouteloua*	4.95 ± 0.54^C^

**Table 5:** Absolute and percent changes in number of quadrats occupied by graminoids in 50 resurveyed quadrats on two wet‐mesic prairies in SE Wisconsin between 2007 and 2009. Significance based on Fisher's exact test; ***indicates *P* < 0.001. Species not in text include *Elymus trachycaulus* (Link) Gould ex Shinners, and *Poa pratensis* L.

Faville Prairie	2007	2009	% change	*P*	Species
	44	0	–100	6.44E‐22***	*Sporobolus heterolepis*
	23	1	–96	1.38E‐07***	*Schizachyrium scoparium*
	35	9	–74	2.50E‐07***	*Sorghastrum nutans*
	12	29	+142	1.02E‐03***	*Andropogon gerardii*
	2	6	+200	0.269	*Spartina pectinata*
	2	30	+1400	8.29E−10***	*Carex* spp.
	0	9	+∞	2.63E‐03**	*Calamagrostis canadensis*
	0	31	+∞	1.10E−10***	*Eleocharis compressa*
**Snapper Prairie**					
	35	0	–100	4.11E‐15***	*Sorghastrum nutans*
	18	0	–100	1.18E‐06***	*Schizachyrium scoparium*
	14	0	–100	4.24E‐05***	*Agrostis gigantea*
	13	0	–100	9.98E‐05***	*Sporobolus heterolepis*
	1	0	–100	0.999	*Elymus trachycaulus*
	6	1	–83	0.111	*Poa pratensis*
	4	4	0	1.000	*Andropogon gerardii*.
	6	8	+33	0.774	*Spartina pectinata*
	5	24	+380	4.66E‐05***	*Carex* spp.
	0	36	+∞	9.49E‐16***	*Digitaria cognata*

***P < 0.001.

## DISCUSSION

Our five study species replaced each other as dominant C_4_ grasses along Curtis’ (1959) gradient from dry to wet prairies in the Upper Midwest (Table 1), with similar patterns seen in eastern Nebraska (White and Glenn‐Lewin, 1984) and Kansas (Heckathorn and DeLucia, 1994) throughout much of the tallgrass prairie biome. We sought to test whether the different distributions of these dominants—with *Bouteloua curtipendula* and *Schizachyrium scoparium* common on drier sites, *Sorghastrum nutans* and especially *Spartina pectinata* common on wetter sites, and *Andropogon gerardii* distributed widely—reflect differences among species in their physiological tolerance of flooding, using three lines of evidence from a greenhouse experiment, a mesocosm experiment, and a natural experiment, with the latter two affected by record rainfalls and consequent flooding.

First, the greenhouse experiment and especially the mesocosm experiment clearly showed that *Bouteloua curtipendula* cannot tolerate flooded conditions (Tables 2–4). The short stature of *B. curtipendula* relative to other C_4_ dominants implies that competition should also help exclude it from wetter, more productive, more densely covered prairies (Givnish, 1982, 1995; Tilman, 1988). Data from other studies suggest that competition may play a role in excluding *Bouteloua* from mesic prairies. For example, Dokken and Hulbert (1978) found that it nearly disappeared during 3 years of fire suppression on a deep‐soil prairie at KPBS while the much taller *Andropogon gerardii* accumulated many tall dead stems and shaded shorter species. The importance of competition in excluding *Bouteloua* from more productive sites is also consistent with the findings that, in mesic prairies, *Bouteloua* is more abundant where grazers suppress the dominant, taller grasses (Hartnett et al., 1996; Fahnestock and Detling, 2002). Given the speed with which *Bouteloua* succumbs to wet conditions, it seems unlikely that competition with taller prairie grasses could ever happen fast enough to be decisive in excluding it from wet and wet‐mesic prairies (Table 1). Our finding that *Bouteloua* is flood‐intolerant is novel and strongly suggests that physiological tolerance excludes it from wet and wet‐mesic prairies. Key remaining questions are (1) whether flooding is frequent enough on dry‐mesic and mesic prairies—especially those with a confining layer that could impede drainage following extreme rainfall events, which are becoming much more common in the Midwest (Easterling et al., 2017)—to be important in excluding *Bouteloua* there and (2) whether some aspect of adaptation to dry microsites imposes a trade‐off with flooding tolerance across prairie grasses.

Second, *Spartina pectinata* clearly has an advantage in growth under flooding, based on the results of the mesocosm experiment (Table 2, Figure 2), supporting the Ellenberg hypothesis (hypothesis 1) that context‐dependent advantages in competition may allow it to dominate wet prairies, as observed (Table 1). *Spartina* growth increased by a small but significant amount with increased flooding depth in the mesocosm experiment, while all other species showed small to large declines in growth. Furthermore, all other species all had less biomass in mixtures than in monocultures (Table 2), indicating that interspecific competition had a stronger effect than intraspecific competition. Most likely, the interspecific competitive effect is due to the biomass‐dominant *Spartina*. In the five‐species mixtures and in the wettest of the monocultures, *Spartina* developed more than twice the aboveground biomass of the other four species (Tables 3 and 4). *Spartina* also increased in biomass after the 2007–2008 flooding events in both wet‐mesic prairies, while all grasses adapted to dry‐mesic, mesic, and wet‐mesic prairies collapsed (Table 5). In a greenhouse experiment, Skinner et al. (2009) found that *Spartina pectinata* had the highest flooding tolerance of all grasses tested (including *Andropogon gerardii, Panicum virgatum, Schizachyrium scoparium*, *Sorghastrum nutans*, and the facultative wetland species *Tripsacum dactyloides*), as measured by root length growth under saturated vs. well‐drained conditions. Physiological adaptions to flooding in *Spartina* include abundant aerenchyma. *Spartina*, *Andropogon*, *Sorghastrum*, and *Tripsacum* all had well‐developed aerenchyma in saturated greenhouse pots, but *Spartina* consistently outcompeted those species when flooded in the field (Skinner et al., 2009). *Spartina pectinata* is the only species of those studied here that has been subject to frequent experimental studies of flooding, all of which have found it to be highly flood tolerant, responding positively to continually saturated conditions, able to produce tall shoots, with strong biomass production and low mortality under a variety of flooded conditions (Miller and Zedler, 2003; Kercher and Zedler, 2004a, b; Herr‐Turoff and Zedler, 2007). Rigorous studies of its drought tolerance have yet to be conducted. Its photosynthesis and growth increase with frequent fire (Johnson and Knapp, 1995).

Third, *Schizachyrium scoparium* appears likely to be excluded from wet prairies (Table 1) by its relatively low tolerance of flooding, as shown by plant survival in Faville and Snapper Prairies (Table 5). At both field sites, *Schizachyrium* virtually disappeared after the 2007–2008 flooding events. *Schizachyrium* had the second lowest sensitivity to flooding in the greenhouse experiment (Table 2) but displayed an intermediate level of growth under even the wettest conditions in the mesocosm experiment (Table 3, Figure 2). *Schizachyrium* did show competitive suppression in the mesocosm experiment, presumably due to biomass‐dominant *Spartina* (Table 3) at Cedar Creek, Tilman (1988) found that *Schizachyrium scoparium* was second only to *Andropogon gerardii* in root to shoot biomass ratio at low N treatments, suggesting high competitive ability via resource depletion for nitrate‐N, especially under nutrient‐poor conditions. It is possible that this adaptation may itself lead to lower flooding tolerance, through exposure of extensive fine roots to anoxic conditions.

Fourth, *Sorghastrum* ranked relatively low in survival after flooding in both the greenhouse experiment and the natural experiment (Tables 2 and 5) and after competitive suppression in the mesocosm experiment, suggesting that physiological tolerance and competition may be limiting its abundance in wet prairies (Table 1). Despite clear dominance in some frequently burned dry to dry‐mesic prairies in the Upper Midwest, both *Schizachyrium* and *Sorghastrum* are not infrequent in wet mesic prairies (Table 1; Bowles and Jones, 2007), and even calcareous seeps (Bowles et al. 2005), suggesting that both share a relative broad tolerance of wetter conditions. In valleys of tallgrass prairies in western Iowa and eastern Nebraska, Weaver and Fitzpatrick (1932) claimed that *Sorghastrum* was favored by flooding and disturbance.

Finally, *Andropogon* showed the highest tolerance of flooding among the four species tested in the greenhouse experiment (Table 2). Its tolerance of flooding was significantly greater than that of *Bouteloua* and *Schizachyrium*, with an LD_50_ exceeding that of the two other species by 51 to 180%. *Andropogon* also more than doubled in abundance at Faville Prairie following the 2007–2008 flood events, though its occurrence at Snapper Prairie did not change (Table 5). Although *Andropogon gerardii* is a regular but minor constituent of wetland communities (Table 1; Swink, 1994), it generally has not been tested for flood tolerance. *Andropogon gerardii* can survive up to 17 weeks of flooding in mesocosms (Kercher and Zedler, 2004a). In Texas and Oklahoma, field studies of 99 sites following flooding, and inundation tests on constructed retention basins, found that *Andropogon* and *Sorghastrum* could tolerate up to 2 weeks of flooding, but *Schizachyrium* only up to six days (Gamble and Rhoades, 1964).

Across all three experiments, rank in survival or growth under flooding show a relatively high degree of concordance (Table S3: *r*_s_ = 0.8–1.0 for each of three pairwise comparisons, *P* < 0.17 to *P* < 0.05; doi:10.5061/dryad.m37pvmd8t). The mean flood tolerance rank, averaged across all three experiments, identifies *Spartina* as most flood tolerant, *Andropogon* as second most tolerant, *Schizachyrium* and *Sorghastrum* as roughly third most tolerant, and *Bouteloua* clearly the least flood tolerant (Table S3). A drought‐to‐death greenhouse experiment parallel to our flood‐to‐death experiment (data not shown) found no significant difference between our four study species in drought tolerance, with the LD_50_ varying from 9.0 ± 4.0 days in *Andropogon* to 14.4 ± 2.2 days in *Schizachyrium*. The fact that *Andropogon* clearly had flooding tolerance—and did not differ significantly from any of the species tested in drought tolerance—may account for its broad distribution across the prairie moisture gradient. It may also help account for *Andropogon* dominating large areas across the Great Plains (Bachle et al., 2018). Given its very high root to shoot ratio in low soil N, *Andropogon* may partition resources not so much by moisture or correlated conditions, as by depleting soil nitrate‐N availability (Tilman, 1988).

Other things being equal, we might expect that closely related grass species might be ecologically and functionally most similar. *Andropogon*, *Schizachyrium*, and *Sorghastrum* share a most recent common ancestor (MRCA) from 9.5 million years ago (Mya) (Welker et al., 2020); *Bouteloua* and *Sporobolus* are more distantly related, with an MRCA of 22.7 Mya, and all five grasses have a MRCA of 47.2 Mya (Givnish et al., 2018). But 5–10 My can lead to enormous ecological divergence in many groups (e.g., Hawaiian silverswords [Landis et al., 2018], Hawaiian lobeliads [Givnish et al., 2009], Andean *Lupinus* [Hughes and Atchison, 2015]). The past 9.5 My has led to substantial divergence among our three most closely related species in ecological distribution (dry‐mesic, mesic, and wet‐mesic prairies), LD_50_ for tissue survival in this study (59% of the total observed range vs. 20% of that in MRCA), 67% of the variation across all species in the leaf water potential at which stomatal close completely (Heckathorn and DeLucia, 1994), and roughly half the leaf height in the range of prairie communities they dominate (Wernerehl and Givnish, 2015). So phylogenetic conservatism does not appear to be an important factor shaping our findings.

The fundamental distinction among our studies is the contrast of lab experiments vs. mesocosm experiments vs. “natural experiments”, with these last involving responses to exceptionally heavy rains and flooding in natural prairies. The lab experiments involved monocultures; the mesocosm experiments involved comparisons among monocultures, among mixtures, and between monocultures and mixtures for individual species; the natural experiments involved responses of individual species to flooding in multispecies prairies. Rarely are lab, mesocosm, and “natural” experiments all used to investigate the ecological causes of differences in species distributions.

To summarize, we found strong experimental support for differences among dominant C_4_ prairie grasses in flooding and drought tolerance, bolstered by field data supporting differential flooding tolerance, supporting hypothesis 3. We also found moderate support from mesocosm experiments for the impact of competition in shaping species distributions under wet conditions, pointing to the apparent competitive effect of *Spartina* on the other species, supporting hypothesis 1 in part. Extremely heavy rains during the mesocosm experiment, however, made it impossible to assess competitive effects among species along an extended moisture gradient for a more comprehensive test of hypothesis 1. Smith et al. (2023) recently used multiple common‐garden experiments on ten species of *Eucalyptus* that dominate different portions of a climatic moisture gradient in Australia to test hypothesis 1 and found, as predicted, that the species with the highest realized height growth in isolation under a given climate were those that dominated the natural vegetation nearby. Hypothesis 2—that species distributions are determined by the minimum levels to which they can reduce soil moisture—remains untested by this or any other study.

This study provides a first step toward understanding the differential distribution of dominant C_4_ grasses along the prairie moisture continuum in the Upper Midwest. The importance of flooding in shaping species abundances in low‐lying prairies is almost surely going to continue to increase in coming years. Extreme rain events (99th percentile) in the Midwest increased by 42% from 1958 to 2016 (Easterling et al., 2017) and are expected to increase by another 40% by 2070–2099 (Feng et al., 2016). Such events will almost surely lead to a doubling or more of flooding of low‐lying prairies since the 1950s; such events have important implications for prairie conservation and restoration moving forward.

Future research needs on the causes of the differential distribution of dominant C_4_ grasses in tallgrass prairies include (1) greenhouse measurements of drought tolerance of *Spartina pectinata*, (2) additional mesocosm studies in which depth to water table or total rainfall is controlled via rain‐off shelters to better assess the role of growth rate, competition, and physiological tolerance in shaping species distributions (i.e., test hypothesis 1 as originally planned in this study); and (3) explore the intriguing proposal by O'Keefe and Nippert (2017) that dominant C_4_ grasses may partly gain an advantage over other growth forms by depleting soil water supplies via nighttime transpiration. Given that soil cation supplies and mechanical impedance appear to be even more strongly related to species composition than moisture availability along Curtis’ prairie continuum (Wernerehl and Givnish, 2015), experiments that (4) separate the effects of moisture, cation supply, and mechanical impedance might be particularly illuminating.

## AUTHOR CONTRIBUTIONS

R.W.W. and T.J.G. conceptualized and designed the study. R.W.W. carried out the experiments and fieldwork, analyzed the data, and wrote the first draft of the manuscript. R.W.W. and T.J.G. edited later drafts of the manuscript.

## Supporting information


Appendix S1.
**Table S1.** See https://doi.org/10.5061/dryad.m37pvmd8t.
**Table S2.** Two‐way ANOVA of square‐root transformed data on aboveground biomass from mesocosm monocultures as a function of species and water‐table depth treatment.
**Table S3.** Ranking of species by flooding tolerance based on survival or growth in the greenhouse, mesocosm, and field studies.

## Data Availability

Raw data (Table S1), code, input data for code, and explanation for the code supporting our analyses are available at Dryad (https://doi.org/10.5061/dryad.m37pvmd8t).
