# Supplementary material for: Flood‐driven survival and growth of dominant C4 grasses helps set their distributions along tallgrass prairie moisture gradients
Source: Am J Bot. 2025 Jan 8;112(1):e16457. doi: 10.1002/ajb2.16457 (PMC11744443; doi:10.1002/ajb2.16457)
Supplement: Supplementary file 1 — Appendix S1. Table S1. See https://doi.org/10.5061/dryad.m37pvmd8t. Table S2. Two‐way ANOVA of square‐root transformed data on aboveground biomass from mesocosm monocultures as a function of species and water‐table depth treatment. Table S3. Ranking of species by flooding tolerance based on survival or growth in the greenhouse, mesocosm, and field studies. [file AJB2-112-e16457-s001.pdf]

ONLINE APPENDIX

Flood-driven survival and growth of dominant C_4 grasses helps set their distributions along tallgrass prairie moisture gradients

Robert W. Wernerehl[1] and Thomas J. Givnish[2]

[1]State Botanist, Massachusetts Natural Heritage and Endangered Species Program, Westborough, MA 01581

[2]Department of Botany, University of Wisconsin-Madison

Madison, WI 53706

Table S1. See https://doi.org/10.5061/dryad.m37pvmd8t.

Table S2. Two-way ANOVA of square-root transformed data on aboveground biomass from mesocosm monocultures as a function of species and water-table depth treatment.

Source of variation	SS	df	MS	F ratio	P-value
Treatment	21.12	4	5.28	5.08	<0.00050
Species	260.82	4	65.20	62.73	<0.00001
Treatment x species	101.23	16	6.33	6.09	<0.00001
Within	623.63	600	1.04		
Total	1006.81	624			

Table S3. Ranking of species by flooding tolerance based on survival or growth in the greenhouse, mesocosm, and field studies. Species with a high rank (smaller number) had high survival in the experiment indicated.

Species	Greenhouse flood rank	Mesocosm flood rank	Field flood rank	Mean flood rank
Spartina	--	1	1	1.00
Andropogon	1	2	2	1.67
Schizachyrium	3	3	3	3.00
Sorghastrum	2	4	4	3.33
Bouteloua	4	5	5*	4.50

*Inferred from absence from wet-mesic prairies
